# Integration of Bioglass Into PHBV-Constructed Tissue-Engineered Cartilages to Improve Chondrogenic Properties of Cartilage Progenitor Cells

**DOI:** 10.3389/fbioe.2022.868719

**Published:** 2022-05-23

**Authors:** Ke Xue, Shuqi Zhang, Jin Ge, Qiang Wang, Lin Qi, Kai Liu

**Affiliations:** ^1^ Department of Plastic and Reconstructive Surgery, Shanghai 9th People’s Hospital, Shanghai Jiao Tong University School of Medicine, Shanghai, China; ^2^ Department of Burn and Plastic Surgery, Hainan Western Central Hospital, Hainan, China; ^3^ Chongqing Key Laboratory of Oral Diseases and Biomedical Sciences, Chongqing Municipal Key Laboratory of Oral Biomedical Engineering of Higher Education, Stomatological Hospital of Chongqing Medical University, Chongqing, China; ^4^ Shanghai Key Laboratory of Stomatology and Shanghai Research Institute of Stomatology, Department of Oral Surgery, Shanghai Ninth People’s Hospital, College of Stomatology, Shanghai Jiao Tong University School of Medicine, Shanghai, China; ^5^ Liaoning Provincial Key Laboratory of Oral Diseases, School and Hospital of Stomatology, China Medical University, Shenyang, China; ^6^ Department of Radiology, Huadong Hospital Affiliated to Fudan University, Shanghai, China

**Keywords:** cartilage progenitor cells, PHBV, Bioglass, hydrophilicity, cartilage engineering

## Abstract

**Background:** The Poly (3-hydroxybutyrate-co-3-hydroxyvalerate) (PHBV) scaffold has proven to be a promising three-dimensional (3D) biodegradable and bioactive scaffold for the growth and proliferation of cartilage progenitor cells (CPCs). The addition of Bioglass into PHBV was reported to increase the bioactivity and mechanical properties of the bioactive materials.

**Methods:** In the current study, the influence of the addition of Bioglass into PHBV 3D porous scaffolds on the characteristics of CPC-based tissue-engineered cartilages *in vivo* were compared. CPCs were seeded into 3D macroporous PHBV scaffolds and PHBV/10% Bioglass scaffolds. The CPC–scaffold constructs underwent 6 weeks *in vitro* chondrogenic induction culture and were then transplanted *in vivo* for another 6 weeks to evaluate the difference between the CPC–PHBV construct and CPC–PHBV/10% Bioglass construct *in vivo*.

**Results:** Compared with the pure PHBV scaffold, the PHBV/10% Bioglass scaffold has better hydrophilicity and a higher percentage of adhered cells. The CPC–PHBV/10%Bioglass construct produced much more cartilage-like tissues with higher cartilage-relative gene expression and cartilage matrix protein production and better biomechanical performance than the CPC–PHBV construct.

**Conclusion:** The addition of Bioglass into 3D PHBV macroporous scaffolds improves the characteristics of CPC-based tissue-engineered cartilages *in vivo*.

## Introduction

Cartilage defects caused by trauma, tumors, and degenerative diseases are becoming increasingly popular, which resulted in significant morbidity and pain over time. Cartilage regenerative medicine and cartilage tissue engineering provide a new and more effective treatment option for the repair of cartilage deficiencies (Hacken et al., 2020). Seeding isolated chondrocytes, mesenchymal stem cells, or cartilage progenitor cells on three-dimensional (3D) biodegradable scaffolds to produce tissue-engineered cartilages is a promising method in cartilage tissue engineering and cartilage regenerative medicine (Kwon et al., 2019).

Autologous chondrocytes is the first option of seeding cells. Vacanti et al. reported the regeneration of nasoseptal cartilage replacements constructed by biodegradable polymers and chondrocytes (Puelacher et al., 1994). Kyoung-Ho Yoon et al. reported that autologous costal chondrocyte implantations can be used as a promising treatment option for repairing articular cartilage defects with good structural regeneration and clinical outcomes and with stable results at midterm follow-up (Yoon et al., 2020). Ning Ma et al. used the tissue-engineered cartilage constructed by autologous chondrocytes and allogeneic, acellular cartilage matrices to repair the cartilage defects (Ma et al., 2017). However, cartilage tissue engineering needed a large number of cells, while chondrocyte expansion *in vitro* led to aging and loss of the chondrocyte phenotype (Thompson et al., 2017).

Bone marrow-derived stem cells (BMSCs) were considered to be promising seeding cells due to their multipotent differentiation ability toward osteogenesis, adipogenesis, and chondrogenesis and high proliferation ability (Fu et al., 2019). Liu et al. reported that BMSC combined with the PRP scaffold differentiated into cartilage tissues and may be a promising therapeutic option for the repair of cartilage defects (Liu et al., 2019). Xue et al. indicated that acellular cartilage sheets could efficiently repair articular cartilage defects by promoting endogenous chondrogenesis *in situ* or inducing chondrogenic differentiation of BMSCs (Xue et al., 2018a). However, it is reported that BMSCs underwent “dedifferentiation” and “phenotypic loss” during *in vitro* expansion and the chondrogenic differentiation process (Vinardell et al., 2012).

In our previous studies, we found that cartilage progenitor cells (CPCs) could be promising alternative cell sources in cartilage tissue engineering and regenerative medicine, and the CPC–poly (3-hydroxybutyrate-co-3-hydroxyvalerate) (PHBV) constructs could form ivory-whitish cartilage-like tissues, with typical cartilage structures (Xue et al., 2019). However, PHBV has weak surface hydrophilic properties, which led to a low number of adhered cells.

Bioglass is a bioactive inorganic material consisting of CaO, Na_2_O, SiO_2_, and P_2_O_5_ in certain proportions. 45S5 is the original component of Bioglass (Islam et al., 2022). It has been reported that the addition of 45S5 Bioglass into PHBV can increase the hydrophilicity of the biomaterials. Therefore, PHBV and PHBV/10% Bioglass (45S5) 3D biomaterial scaffolds were prepared in this study, CPCs were combined with two different scaffolds and incubated *in vitro* for 6 weeks, and then subcutaneous transplantation was performed for another 6 weeks. The cell adhesion, production of the extracellular matrix, size, structure, and functional and biomechanical characteristics of the regenerated cartilage were determined to analyze the influence of the addition of 10%Bioglass into the PHBV scaffold on the function and structure of the neocartilage.

## Materials and Methods

All animal experimental procedures and operation in the current research have been approved by the Ethics Committee of Shanghai 9th People’s Hospital, Shanghai Jiao Tong University School of Medicine (SH9H-2021-A416-SB).

### Preparation of PHBV and PHBV/10%Bioglass Composite Scaffolds

PHBV (Mw = 300,000), consisting of 3 mol% 3-hydroxyvalerate units, was purchased from Tianan Biologic Material Co., Ltd. (Ninbo, China). A solvent casting/particulate leaching method was used to produce PHBV and PHBV/10%Bioglass bioactive composite scaffolds as reported previously (Aboudzadeh et al., 2021). Briefly, the dissolution of 1 g of PHBV powder into 10 ml of chloroform produced a concentration of 10% (w/v), and then Bioglass powder (0.125 g) was dissolved into the mixture to obtain the PHBV/10%Bioglass composite scaffolds. After the sodium chloride (NaCl) particles were mixed into the above solution as porogens, the mixture was transferred to a Teflon mold (inner diameter 80 mm, height 2 mm). The samples were air-dried for 24 h to remove the solvent and then were vacuum-dried for 48 h at 60°C to evaporate any remaining water-insoluble solvent.

The NaCl (porogens) in the dried scaffold was leached out by immersing in deionized water and then was vacuum-dried to produce porous 3D bioactive scaffolds. The scaffolds were prepared in the shape of a cylinder (5 mm side diameter, 2 mm thick) in the current research.

### Property of the PHBV and PHBV/10%Bioglass Scaffolds

Optical microscopy and scanning electron microscopy (SEM) were used to evaluate the difference between the two kinds of scaffolds. ImageJ software was used to analyze/process the SEM images of the scaffolds to obtain the porosity and pore size distribution data. The mass of the scaffolds and dimensions were measured to analyze the porosity ratio of the 3D porous bioactive scaffolds as previously described (El-Shanshory et al., 2022). The compressive strength of the 3D bioactive scaffolds was determined according to the force–displacement curve with a Shimadzu AG mechanical tester (Wright et al., 2022) (Shimadzu Co., Japan).

### Hydrophilicity, Water Absorption, and Cell Adhesion Determination

The water contact angles of the nonporous PHBV and PHBV/10% Bioglass composite cuboids were evaluated to determine the hydrophilicity of the two kinds of scaffold composites. The sessile drop technique was used to evaluate the water contact angles at 25°C using a contact angle measuring instrument (SZ10-JC2000A, Shanghai, China) (Yang et al., 2020).

The water absorption of the two kinds of 3D scaffolds was tested according to the protocol described previously (Tan et al., 2022). The weights of completely dried PHBV and PHBV/10%Bioglass bioactive scaffolds were measured (W dry), and then they were immersed in deionized water to achieve water absorption equilibration for 4 h at 25 °C. Then, the weight of hydrated 3D scaffolds was measured (W wet), and the water absorptivity was determined according to the formula

ratio (%) = (Wwet - Wdry)/Wdry×100%.

The percentage of adhered cells was determined by 3-(4,5-dimethylthiazol-2-yl)-2,5-diphenyltetrazonium bromide (MTT)-based colorimetric assay as previously described (Xue et al., 2019). Briefly, the CPCs at passage 2 were harvested and seeded onto the sterilized PHBV and PHBV/10%Bioglass composite substrates and then cultured in a CO_2_ incubator for 4 h. Then, 1 ml of fresh low-glucose Dulbecco’s modified Eagle’s medium (DMEM) was dropped into each well, and the MTT method was used to measure the number of living cells.

### Cell Harvesting and Construction of Tissue-Engineered Cartilages

The differential adhesion to the fibronectin method was used to obtain CPCs from chondrocytes. The harvested articular cartilage mass was cut into (1–2) mm^2^ fragments and then washed with sterile chloromycetin and phosphate-buffered saline (PBS) thrice. The minced cartilage fragments were digested in a solution of collagenase II (0.2% w/v) in high-glucose DMEM, then were filtered with a 200 μm filter to remove undigested tissues, and then were subjected to cell suspension. 5,000 cells/ml were plated onto 100 mm dishes (prior treated with 10 μg/ml fibronectin for 24 h) at 37°C for 20 min in a Thermo Scientific™ CO_2_ incubator. The nonadherent cells were removed after 20 min and washed with PBS twice, and then 10 ml of low-glucose DMEM with 10% fetal bovine serum (FBS) was dropped into each dish. The remaining cells were incubated for 7–10 days until the cell confluence reached 80%. Then, the cells were subcultured at a density of 3×10^4^ cells/cm^2^.

The cylindrical PHBV scaffold and PHBV/10%Bioglass scaffold were sterilized and placed in the center of six-well polystyrene culture plates. 30 µl (5 × 10^7^ cell/ml) of the CPC suspension at passage 2 was seeded onto the 3D bioactive scaffold and inoculated at 37°C for 4 h. This allowed adequate attachment of the CPCs onto the 3D bioactive scaffolds. Then, 5 ml of low-glucose DMEM with 10% FBS was dropped into each well after 4 h, and the culture medium was refreshed every 2 or 3 days. The athymic C57BL/6 nude mice were anesthetized intraperitoneally with sodium pentobarbital (60 mg/kg); then the cell-scaffold constructs after 6-week *in vitro* culture were transplanted into the subcutaneous tissue of the back of the mice for another 6 weeks, and then the specimens were harvested.

### Cell Proliferation

The DNA content of the samples was tested to determine the number of CPCs on the scaffolds after being *in vitro* cultured for 1 week and 2 weeks (Luo et al., 2021). The cell proliferation on the scaffolds was assessed via the MTT assay, and the CPCs were incubated for 1 day, 3 days, and 5 days and then tested with the MTT method.

### Chondrogenic Induction *in Vitro*

After 3 days of incubation in low-glucose DMEM composed of 10% FBS, the regular culture medium was refreshed with a chondrogenic induction medium containing high-glucose DMEM containing 10% FBS supplemented with 50 ng/ml insulin-like growth factor 1 (IGF-1, Peprotech, Rocky Hill, NJ), 10 ng/ml transforming growth factor β1 (TGF-β1, Peprotech, Rocky Hill, NJ), and 40 ng/ml dexamethasone (Sigma, St. Louis, MO). The culture medium change was performed every 3 days.

### Characterization of *in Vivo* Tissue-Engineered Cartilages

#### Gross Evaluation of *in Vivo* Tissue-Engineered Cartilages

After 6 weeks of subcutaneous implantation, the thickness and diameter of the cell–scaffold construct were measured using a vernier caliper, and the volume of the cell–scaffold construct was determined using a volumenometer.

#### Quantitative Evaluation of *in Vivo* Tissue-Engineered Cartilages

The wet weight, total collagen content (Guedes et al., 2022), and glycosaminoglycan (GAG) content (Nunes et al., 2021) of the specimen after 6 weeks of subcutaneous transplantation were determined using the protocol previously reported. The biomechanical testing was tested using a biomechanical analyzer according to the previous protocol, and the force–displacement curve was used to calculate the compression strength of the cell-scaffold construct (Wright et al., 2022).

#### Histological Evaluation

The specimen after subcutaneous implantation for 6 weeks was immersed in 10% neutral buffered formalin, washed with PBS, dehydrated, embedded in paraffin, cut into slices of a 5 μm thickness, and stained with hematoxylin and eosin. 5 μm slices were immunostained with a type II collagen antibody as previously described (Lv et al., 2020).

### RT-PCR Analysis

Total RNA was extracted from the specimen, and cDNA was harvested by reverse transcription (RT) using previous protocols (Xue et al., 2018b). Real-time quantitative polymerase chain reaction (RT-PCR) was used to analyze the cartilage-specific gene expression: type II collagen (COL II) a1 (Sense 5′-TGC​TGC​TGA​CGC​TGC​TC-3′, Antisense 5′-GTT​CTC​CTT​TCC​TGT​CCC​TTT​G-3′), SOX-9 (Sense 5′-GGC​TCG​GAC​ACA​GAG​AAC​AC-3′, Antisense 5′-GTG​CGG​CTT​ATT​CTT​GCT​CG-3′), and aggrecan (Sense 5′-GGG​GAA​TCT​TCT​GGC​ATT​AA-3′, Antisense 5′-CGT​TGG​AGC​CTG​GGT​T-3′). The β-actin (Sense 5′-ACA​TCA​AGG​AGA​AGC​TCT​GCT​ACG-3′, Antisense 5′-GAG​GGG​CGA​TGA​TCT​TGA​TCT​TCA-3′) mRNA level was used as an internal control.

### Statistical Analysis

All harvested data were expressed as the means ± standard deviation (*n* = 6). The data differences between the PHBV and PHBV/10%Bioglass bioactive scaffolds were evaluated by Student’s t-test. A *p*-value less than 0.05 was considered to be statistically significant.

## Results

### Optical Microscopy and SEM of PHBV/10%Bioglass Scaffolds and the CPCs–PHBV/10%Bioglass Construct

**FIGURE 1:**
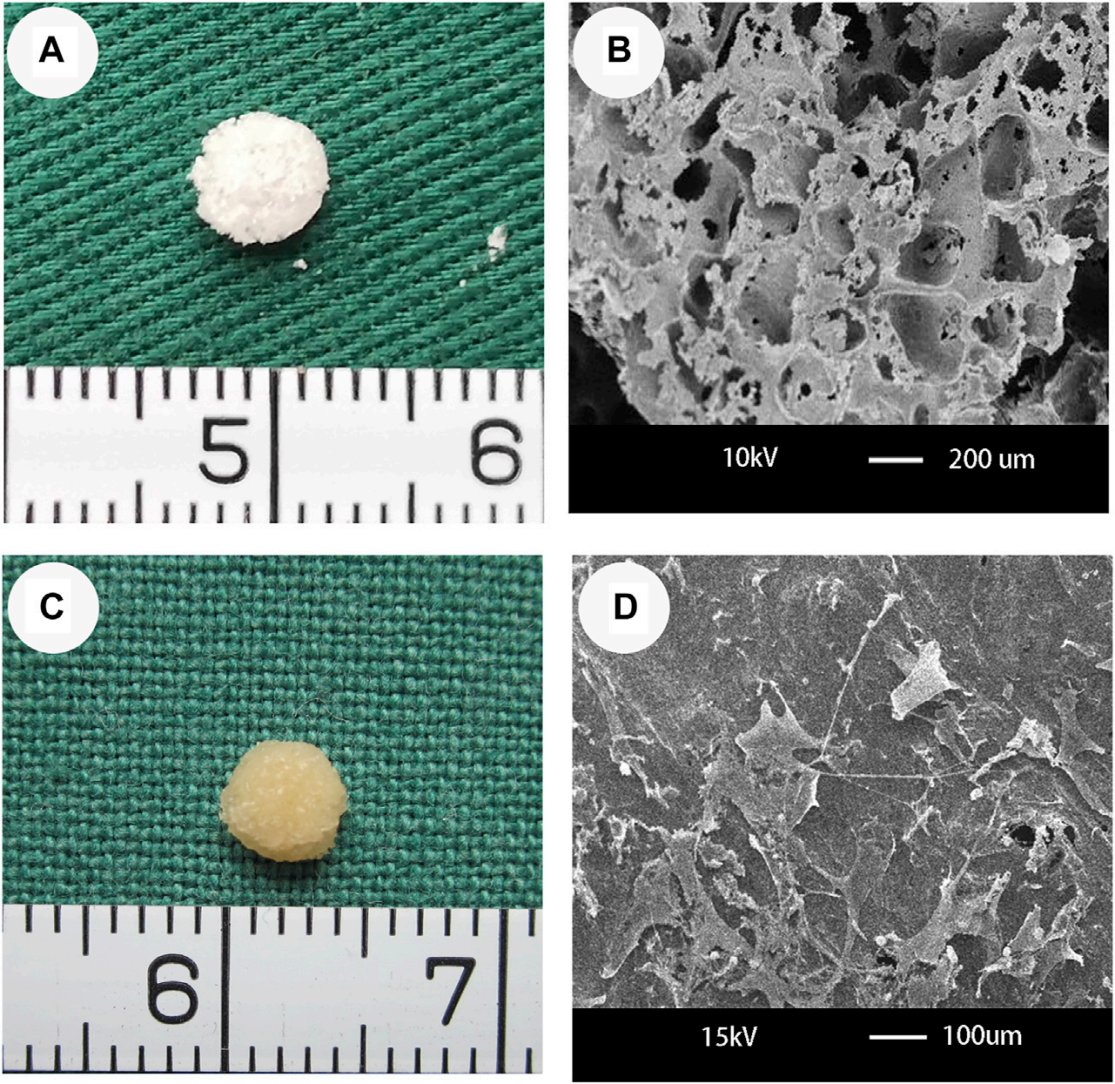
Optical microscopy and SEM of PHBV/10%Bioglass scaffolds and the CPC–PHBV/10%Bioglass construct. **(A)** The PHBV/10%Bioglass scaffolds exhibited a cylindrical shape (5 mm diameter and 2 mm thickness), with a lot of pores on the surface of the composite scaffolds. **(B)** PHBV/10%Bioglass composite porous 3D scaffolds had a macroporous structure with interconnected open pores of 30–300 μm in diameter. **(C)** Gross view of *in vitro* CPC–scaffold constructs after 6 weeks of *in vitro* culture. These engineered tissues roughly maintained their original cylindrical shape and size and exhibited an ivory-whitish appearance. **(D)** SEM view of CPCs-PHBV/10%Bioglass constructs after 6 weeks of *in vitro* culture, exhibiting abundant extracellular matrix production and good compatibility of the CPCs with the composite scaffold.

The PHBV/10%Bioglass scaffolds exhibited a cylindrical porous scaffold shape (Figure 1A). PHBV/10%Bioglass composite scaffolds show a three-dimensionally interconnected macroporous structure with the pore diameter distribution varying from 30 to 300 μm (Figure 1B). A gross view of *in vitro* CPC–scaffold constructs after culture for 6 weeks shows that these engineered tissues maintained their original sizes roughly and exhibited a yellowish appearance (Figure 1C). The SEM view of cell–PHBV/Bioglass constructs after 6 weeks of *in vitro* culture shows that the CPCs adhered to the scaffold pore walls and distributed throughout the scaffold pores homogeneously, showed an extended morphology, and exhibited abundant extracellular matrix production and good compatibility of the CPCs with the PHBV/10%Bioglass (Figure 1D).

### Properties of PHBV and PHBV/10%Bioglass Bioactive Scaffolds

**FIGURE 2:**
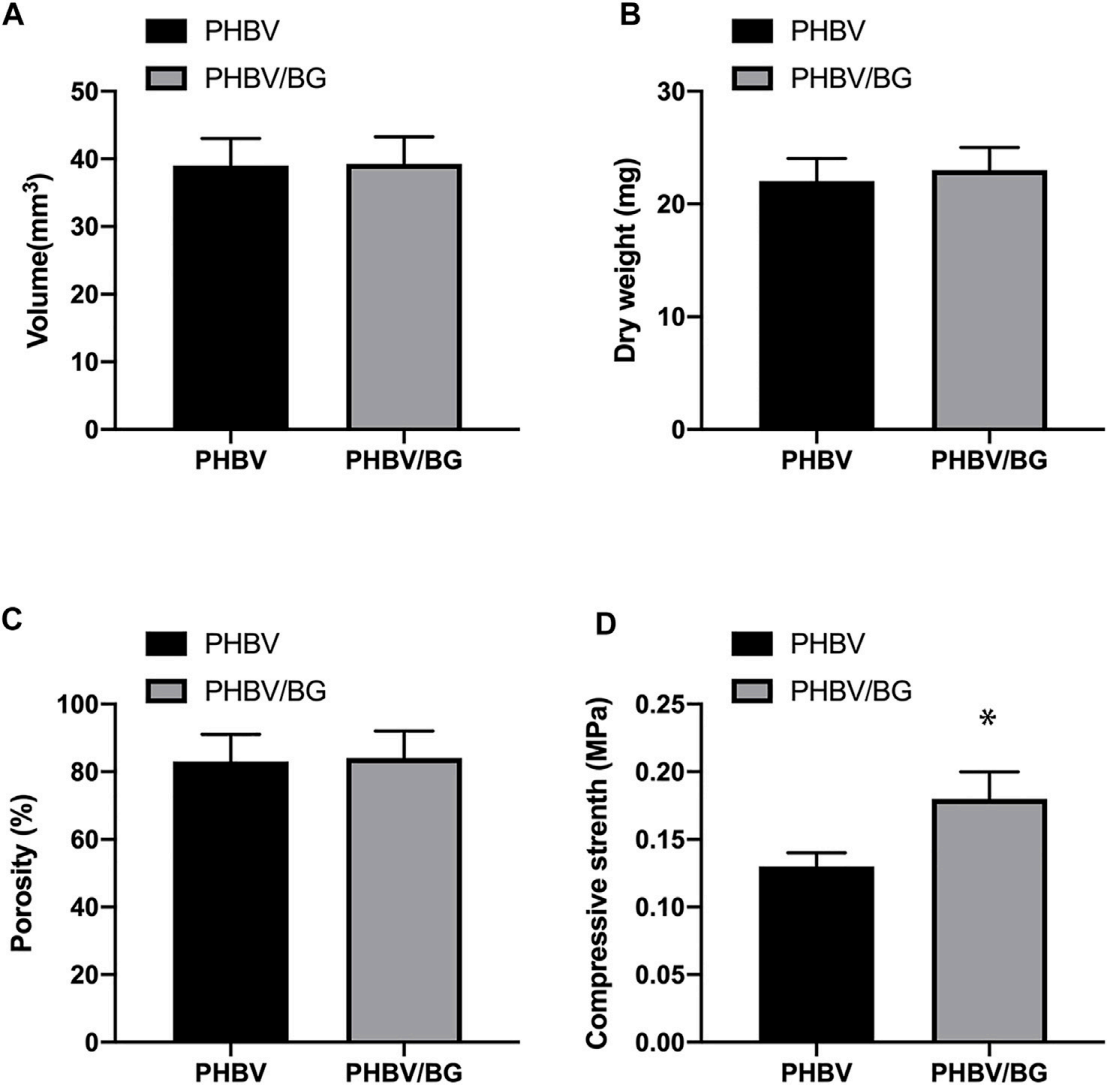
Characterization of PHBV and PHBV/10%Bioglass 3D porous scaffolds. The PHBV and PHBV/10%Bioglass 3D porous scaffolds exhibited the same volume **(A)** and dry weight **(B)** and the same porosity **(C)** (*p* > 0.05). The compressive modulus **(D)** of the CPC–PHBV/10%Bioglass constructs was greater than that of CPC–PHBV constructs (*p* < 0.05).

The PHBV and PHBV/10%Bioglass 3D scaffolds had similar porosities (*p* > 0.05), and the size of interconnected open pores varied from 30 to 300 μm (shown by SEM analysis). The compressive modulus of the PHBV scaffolds was 0.13 ± 0.01 MPa, while the compressive modulus of the PHBV/10%Bioglass scaffolds was 0.18 ± 0.02 MPa (*p* < 0.05) (Figure 2).

### Contact Angle, Water Absorptivity, and Cell Adhesion of the PHBV and PHBV/10%Bioglass Scaffolds

**FIGURE 3:**
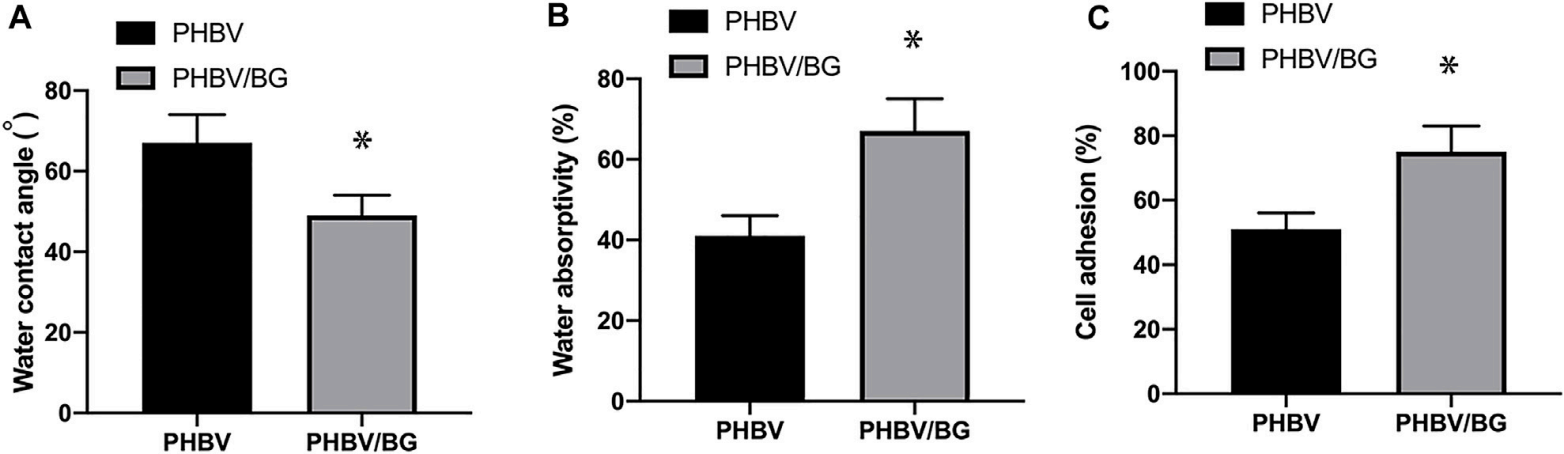
Contact angle, water absorptivity, and cell adhesion of the scaffolds. **(A)** The water contact angles of the PHBV/Bioglass composite scaffolds were significantly lower than that of the pure PHBV scaffold, indicating that there was a significant increase in surface hydrophilicity with the addition of Bioglass into PHBV (*p* < 0.05). **(B)** The water absorptivity of the PHBV/Bioglass composite scaffolds was obviously greater than that of pure PHBV (*p* < 0.05). **(C)** The percentage of adhered cells increased significantly with the addition of Bioglass (*p* < 0.05).

The water contact angle of the PHBV/10%Bioglass is (49 ± 5.1°), while the water contact angle of pure PHBV composites is (67 ± 7.2°) (*p* < 0.05) (Figures 3A,B). The water absorptivity of the PHBV/10%Bioglass is (72 ± 8.1%), while the water absorptivity of pure PHBV composites is (57 ± 6.2%) (*p* < 0.05) (Figure 3B). The percentage of adhered cells of the PHBV/10%Bioglass is (75 ± 7.6%), while the percentage of adhered cells of pure PHBV composites is (51 ± 5.2%) (*p* < 0.05) (Figure 3C).

### Cell Proliferation

**FIGURE 4:**
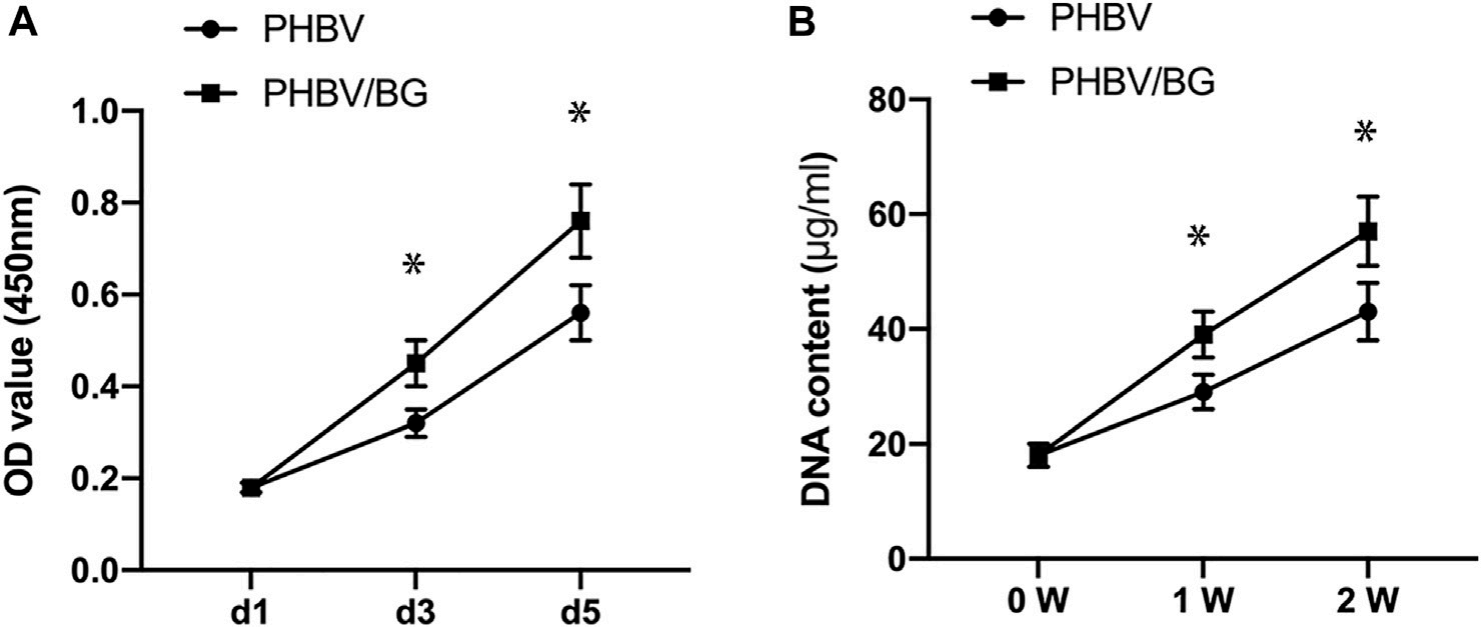
Cell Proliferation. There was an obvious difference in the cellular proliferation between the CPC–PHBV construct and CPC–PHBV/10%Bioglass construct (*p* < 0.05) **(A)**. The DNA content of the CPC–PHBV/10%Bioglass construct is higher than that of the CPC–PHBV construct (*p* < 0.05) **(B)**.

There was an obvious difference in the cellular proliferation of CPC–PHBV constructs and that of CPC–PHBV/10%Bioglass constructs (*p* < 0.05). The DNA content of CPC–PHBV/10%Bioglass constructs is higher than that of CPC–PHBV constructs (*p* < 0.05) (Figure 4).

### Analysis of *in Vivo* Tissue-Engineered Cartilages on PHBV and PHBV/10%Bioglass Scaffolds

#### Gross Analysis of *in Vivo* Tissue-Engineered Cartilages

**FIGURE 5:**
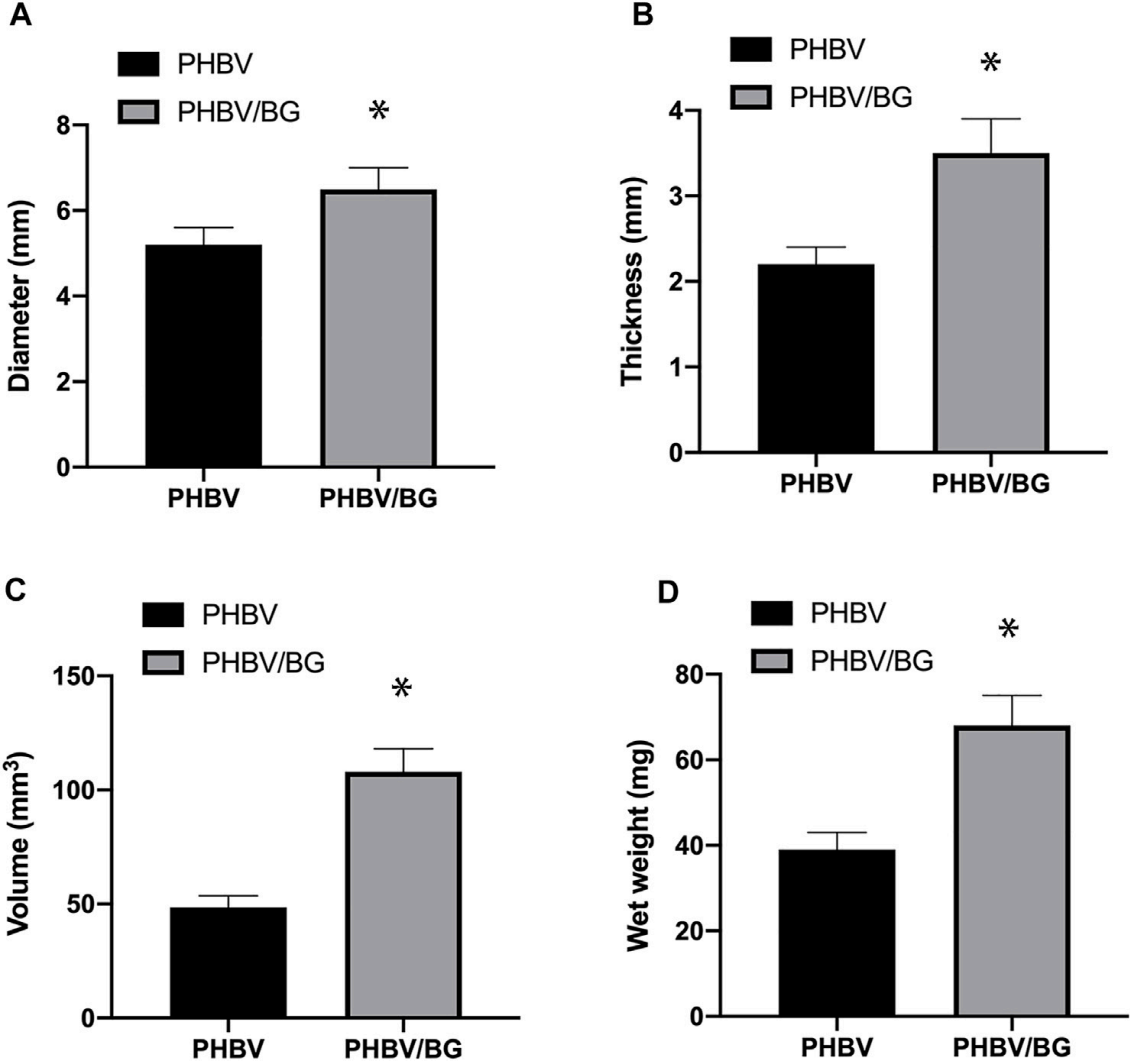
Gross analysis of *in vivo* engineered tissue cartilages. The diameter **(A)**, thickness **(B)**, volume **(C)**, and wet weight **(D)** of the CPC–PHBV/10%Bioglass construct were higher than those of the CPC–PHBV construct *in vivo* (*p* < 0.05).

After subcutaneous transplantation for 6 weeks, the CPC–PHBV constructs and CPC–PHBV/10%Bioglass constructs kept their original cylinder shape and size basically and demonstrated a white cartilage-like appearance. The thickness, diameter, volume, and wet weight of CPC–PHBV/10%Bioglass constructs were more than that of CPC–PHBV constructs (*p* < 0.05) (Figure 5).

#### Histological and Immunohistochemical Evaluation

**FIGURE 6:**
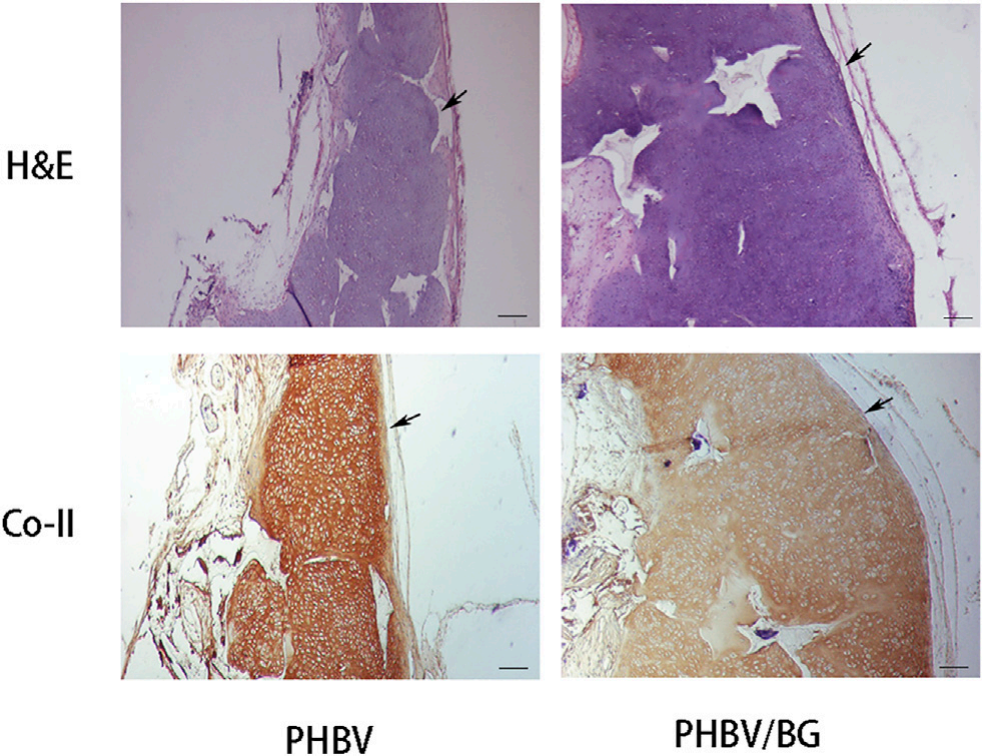
Histological and immunohistological analysis of *in vivo* engineered constructs. There is an obvious difference in the thickness between the CPC–PHBV/10%Bioglass construct and CPC–PHBV construct (*p* < 0.05). The CPC–PHBV/10%Bioglass construct produced much more cartilage-like tissues than the CPC–PHBV construct. Scale bar = 100 μm.

The cartilage-like tissue was produced in both CPC–PHBV constructs and CPC–PHBV/10%Bioglass constructs, with mature cartilage lacuna structure formation and obvious positive type II collagen expression (Figure 6). The histological and immunohistochemical analyses show that the tested specimen produced more cartilage extracellular matrices and created much more cartilage-like tissues in the CPC–PHBV/10%Bioglass constructs than that in CPC–PHBV constructs.

#### Collagen Content, GAG Content, and Compression Strength

**FIGURE 7:**
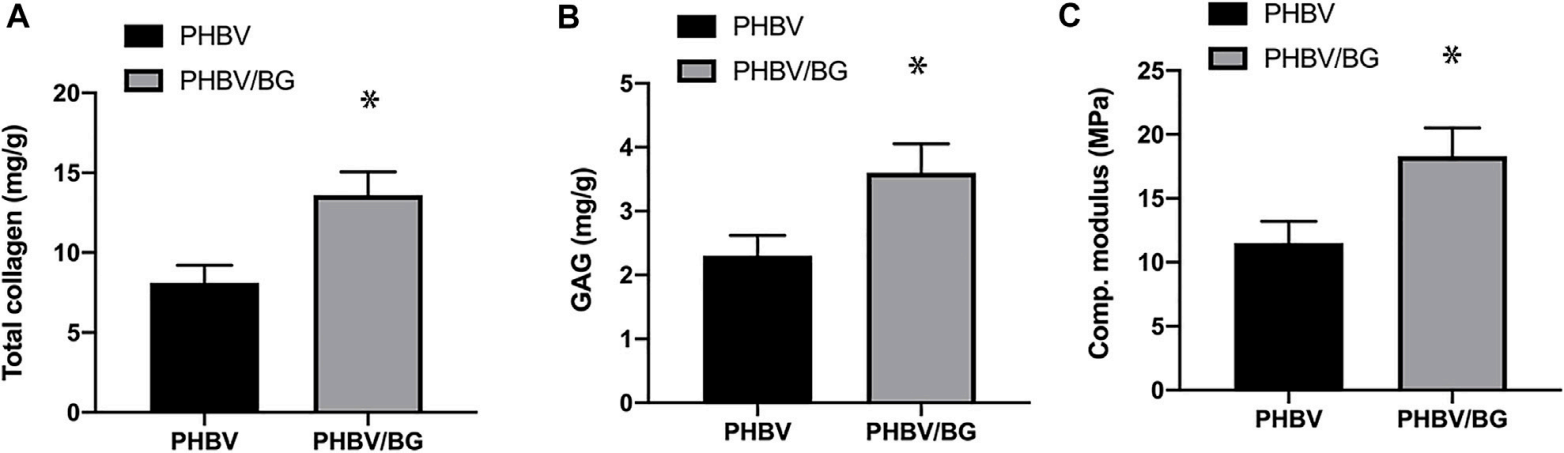
Collagen content, GAG content, and compression modulus. There is a significant difference in terms of collagen **(A)** and GAG **(B)** contents and the compression modulus **(C)** between the CPC–PHBV construct and CPC–PHBV/10%Bioglass construct (*p* < 0.05).

The collagen content of CPC–PHBV/10%Bioglass constructs was 8.1 ± 1.1 mg/g, while the collagen of CPC–PHBV constructs was 13.6 ± 1.45 mg/g (*p* < 0.05). The GAG content of CPC–PHBV/10%Bioglass constructs was 2.3 ± 0.32 mg/g, while the GAG content of CPC–PHBV constructs was 3.6 ± 0.45 mg/g (*p* < 0.05). The compression modulus of CPC–PHBV/10%Bioglass constructs was 11.5 ± 01.7 MPa, while the compression modulus of CPC–PHBV constructs was 18.3 ± 2.2 MPa (*p* < 0.05) (Figure 7).

#### RT-PCR Analysis

**FIGURE 8:**
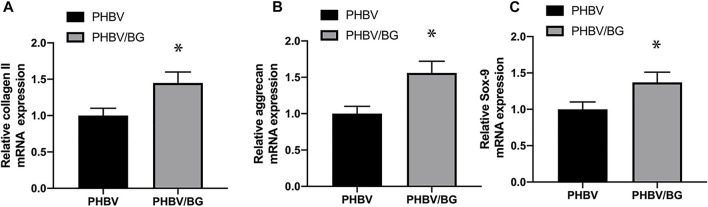
Chondrogenic differentiation of the CPC–PHBV construct and CPC–PHBV/10%Bioglass construct. RT-PCR analysis reveals the stronger expression of COL II **(A)**, aggrecan **(B)**, and the SOX-9 gene of the CPC–PHBV/10%Bioglass construct than the CPC–PHBV construct (*p* < 0.05).

PCR analysis exhibited that aggrecan, collagen II, and SOX-9 of CPC–PHBV/10%Bioglass constructs were all significantly highly expressed compared to CPC–PHBV constructs (*p* < 0.05), indicating that the addition of Bioglass into PHBV may enhance the chondrogenic differentiation of CPCs (Figure 8).

## Discussion

Seed cells, biodegradable scaffolds, and the environment are three key elements in tissue engineering (Antunes et al., 2020). The seed cells on the biomaterial scaffolds should maintain their mature and stable chondrogenic phenotype and produce rich extracellular matrices, which can replace the biodegradable scaffolds eventually and determine the fate of tissue-engineered cartilages (Francis et al., 2018). Due to “dedifferentiation” and “phenotypic loss” during *in vitro* expansion and the chondrogenic differentiation process, bone marrow-derived stem cells (BMSCs) and chondrocytes were not the ideal seeding cells (Shi et al., 2017; Ripmeester et al., 2018). CPCs harvested from cartilage tissues present chondrogenic characteristics and good proliferation ability, thus becoming novel promising seeding cells (Xue et al., 2019).

Due to its appropriate biodegradability and biocompatibility, PHBV has been shown to be a biodegradable biomaterial scaffold used in cartilage tissue engineering and regenerative medicine (Rodrigues et al., 2021). In our previous research, the feasibility of combining CPCs with PHBV to construct tissue-engineered tissues was explored, and we found that the tissue-engineered cartilage on PHBV scaffolds had an insufficient thickness and inadequate biomechanical strength due to the surface hydrophobicity of the scaffold (Xue et al., 2019).

The hydrophilicity is a critical element influencing cell attachment, growth and proliferation, biocompatibility, fast cell adhesion and growth, and physical–chemical resistance (Wang et al., 2022). Hydrophilicity of the material surface can influence cell attachment and cell shape, which can also dictate proliferation and differentiation of cells on the material surface or in the materials (Kunrath et al., 2020). Marcel F Kunrath et al. proposed that the application of plasma-treated surfaces resulted in the most hydrophilic specimen (Kunrath et al., 2020). Some studies have proposed the application of nonthermal atmospheric pressure plasma, and ultraviolet treatments change negatively charged hydrophobic (bioinert) surfaces into positively charged hydrophilic (bioactive) surfaces, improving osteoblastic cell adhesion, albumin adsorption, and cytoskeleton development (Choi et al., 2016). Similarly, UV light has been used to increase hydrophilicity (Ogawa, 2014).

The addition of hydrophilic inorganic substances into hydrophobic materials (PHBV) has been found to be a feasible approach to increase the hydrophilicity of PHBV (Li et al., 2005). Therefore, we investigated the addition of 45S5 Bioglass into PHBV to increase the hydrophilic property of the PHBV scaffold. 45S5 Bioglass is a bioactive glass with remarkable biodegradability and biocompatibility, composed of 24.5 wt% Na_2_O, 45 wt% SiO_2_, 6 wt% P_2_O_5_, and 24.5 wt% CaO (Rizwan et al., 2017). Compared with the pure PHBV 3D porous scaffolds, the addition of 45S5 Bioglass into PHBV has been proven to have better biodegradation, bioactivity, and mechanical properties (Li et al., 2005). Therefore, 10% Bioglass was added into PHBV scaffolds to prepare PHBV/10%Bioglass porous composite scaffolds in current studies.

The water contact angle values are an important measure of the hydrophilicity/hydrophobicity that gives information on the surface properties and wettability of the material surface (Huhtamäki et al., 2018). The superhydrophilic materials (a contact angle less than 10°) will be superhydrophilic with good self-cleaning ability and higher wettability. Similarly, if the contact angle is greater than 150°, the materials will repel water and reduce the water absorption (Yorseng et al., 2020). The water contact angle of PHBV/10%Bioglass composites decreased with the addition of Bioglass. Compared with pure PHBV composites, the water contact angle of the PHBV/10%Bioglass decreased significantly, indicating faster liquid spread over the material surface and better wettability and suggesting that PHBV/10%Bioglass is a more hydrophilic composite biomaterial scaffold. The water absorptivity increased with the addition of Bioglass into PHBV, which indicated that the addition of Bioglass resulted in better wettability and water absorptivity. In addition, the addition of Bioglass did not decrease porosity, the dry weight, the volume, and the structure of the scaffold. The hydrophilicity may increase due to the Bioglass addition, leading to improved cell-adhesion ability. The histological and immunohistochemical staining of the *in vivo* tissue-engineered tissue shows that the CPC–PHBV/10%Bioglass constructs producedmuch more cartilage-like tissues than CPC–PHBV after 6 weeks of subcutaneous implantation.

The compression modulus analysis suggested that tissue-engineered cartilages constructed by PHBV/10%Bioglass scaffolds had better biomechanical properties. On one hand, the addition of Bioglass into PHBV enhanced the mechanical strength of the composite scaffold; the compressive modulus of the PHBV/10%Bioglass composite scaffolds was significantly greater than that of PHBV scaffolds as the PHBV/10%Bioglass composite scaffolds and pure PHBV scaffolds had the same size, volume, and porosity. This indicated that the addition of the Bioglass increased the compressive properties of the 3D composite porous scaffolds significantly. On the other hand, the improved hydrophilicity led to improved cell-adhesion ability. The much more cartilage-like tissues produced by the CPC-PHBV/10%Bioglass construct may be due to increased cell-adhesion ability, leading to the increased compressive strength of the tissue-engineered cartilage.

The extracellular matrix content (GAG and total collagen) determined the mechanical properties of the tissue-engineered cartilage. It was found in our study that the GAG content and the total collagen content of the CPC–PHBV/10%Bioglass construct were significantly greater than those of the CPC–PHBV construct, which also resulted in the increased mechanical strength. In addition, the result of PCR analysis suggested that the addition of Bioglass into PHBV may enhance the chondrogenic differentiation of CPCs.

## Conclusion

The addition of Bioglass into PHBV improved the properties of CPC-based tissue-engineered cartilages *in vivo*, which provide an effective approach for the preparation of 3D porous biodegradable scaffolds with improved bioactivity and mechanical properties for cartilage tissue engineering and cartilage regeneration.

## Data Availability

The original contributions presented in the study are included in the article/Supplementary Material, further inquiries can be directed to the corresponding authors.

## References

[B1] Aboudzadeh N. Khavandi A. Javadpour J. Shokrgozar M. A. Imani M. (2021). Effect of Dioxane and N-Methyl-2-Pyrrolidone as a Solvent on Biocompatibility and Degradation Performance of PLGA/nHA Scaffolds. ibj 25 (6), 408–416. 10.52547/ibj.25.6.408 34641642 PMC8744699

[B2] Antunes A. Popelka A. Aljarod O. Hassan M. K. Kasak P. Luyt A. S. (2020). Accelerated Weathering Effects on Poly(3-Hydroxybutyrate-Co-3-Hydroxyvalerate) (PHBV) and PHBV/TiO2 Nanocomposites. Polymers (Basel) 12 (8), 1743. 10.3390/polym12081743 PMC7464598 32764247

[B3] Choi S.-H. Jeong W.-S. Cha J.-Y. Lee J.-H. Yu H.-S. Choi E.-H. (2016). Time-dependent Effects of Ultraviolet and Nonthermal Atmospheric Pressure Plasma on the Biological Activity of Titanium. Sci. Rep. 6, 33421. 10.1038/srep33421 27627871 PMC5024128

[B4] El-Shanshory A. A. Agwa M. M. Abd-Elhamid A. I. Soliman H. M. A. Mo X. Kenawy E. R. (2022). Metronidazole Topically Immobilized Electrospun Nanofibrous Scaffold: Novel Secondary Intention Wound Healing Accelerator. Polymers (Basel) 14 (3), 454. 10.3390/polym14030454 35160444 PMC8840736

[B5] Francis S. L. Di Bella C. Wallace G. G. Choong P. F. M. (2018). Cartilage Tissue Engineering Using Stem Cells and Bioprinting Technology-Barriers to Clinical Translation. Front. Surg. 5, 70. 10.3389/fsurg.2018.00070 30547034 PMC6278684

[B6] Fu X. Liu G. Halim A. Ju Y. Luo Q. Song A. G. (2019). Mesenchymal Stem Cell Migration and Tissue Repair. Cells 8 (8), 784. 10.3390/cells8080784 PMC6721499 31357692

[B7] Guedes P. L. R. Carvalho C. P. F. Carbonel A. A. F. Simoes M. J. Icimoto M. Y. Aguiar J. A. K. (2022). Chondroitin Sulfate Protects the Liver in an Experimental Model of Extra-hepatic Cholestasis Induced by Common Bile Duct Ligation. Molecules 27 (3), 654. 10.3390/molecules27030654 35163920 PMC8839946

[B8] Hacken B. A. LaPrade M. D. Stuart M. J. Saris D. B. F. Krych A. J. (2020). Small Cartilage Defect Management. J. Knee Surg. 33 (12), 1180–1186. 10.1055/s-0040-1716359 32898908

[B9] Huhtamäki T. Tian X. Korhonen J. T. Ras R. H. A. (2018). Surface-wetting Characterization Using Contact-Angle Measurements. Nat. Protoc. 13 (7), 1521–1538. 10.1038/s41596-018-0003-z 29988109

[B10] Islam M. T. Nuzulia N. A. Macri-Pellizzeri L. Nigar F. Sari Y. W. Ahmed I. (2022). Evolution of Silicate Bioglass Particles as Porous Microspheres with a View towards Orthobiologics. J. Biomater. Appl. 36 (8), 1427–1443. 10.1177/08853282211059294 35050809

[B11] Kunrath M. F. Vargas A. L. M. Sesterheim P. Teixeira E. R. Hubler R. (2020). Extension of Hydrophilicity Stability by Reactive Plasma Treatment and Wet Storage on TiO 2 Nanotube Surfaces for Biomedical Implant Applications. J. R. Soc. Interf. 17 (170), 20200650. 10.1098/rsif.2020.0650 PMC7536041 32993437

[B12] Kwon H. Brown W. E. Lee C. A. Wang D. Paschos N. Hu J. C. (2019). Surgical and Tissue Engineering Strategies for Articular Cartilage and Meniscus Repair. Nat. Rev. Rheumatol. 15 (9), 550–570. 10.1038/s41584-019-0255-1 31296933 PMC7192556

[B13] Li H. Du R. Chang J. (2005). Fabrication, Characterization, and *In Vitro* Degradation of Composite Scaffolds Based on PHBV and Bioactive Glass. J. Biomater. Appl. 20 (2), 137–155. 10.1177/0885328205049472 16183674

[B14] Liu F. Xu H. Huang H. (2019). A Novel Kartogenin-Platelet-Rich Plasma Gel Enhances Chondrogenesis of Bone Marrow Mesenchymal Stem Cells *In Vitro* and Promotes Wounded Meniscus Healing *In Vivo* . Stem Cel Res Ther 10 (1), 201. 10.1186/s13287-019-1314-x PMC6615105 31287023

[B15] Luo L. Foster N. C. Man K. L. Brunet M. Hoey D. A. Cox S. C. (2021). Hydrostatic Pressure Promotes Chondrogenic Differentiation and Microvesicle Release from Human Embryonic and Bone Marrow Stem Cells. Biotechnol. J. 1, e2100401. 10.1002/biot.202100401 34921593

[B16] Lv X. Sun C. Hu B. Chen S. Wang Z. Wu Q. (2020). Simultaneous Recruitment of Stem Cells and Chondrocytes Induced by a Functionalized Self-Assembling Peptide Hydrogel Improves Endogenous Cartilage Regeneration. Front. Cel Dev. Biol. 8, 864. 10.3389/fcell.2020.00864 PMC7493663 33015049

[B17] Ma N. Wang H. Xu X. Wan Y. Liu Y. Wang M. (2017). Autologous-cell-derived, Tissue-Engineered Cartilage for Repairing Articular Cartilage Lesions in the Knee: Study Protocol for a Randomized Controlled Trial. Trials 18 (1), 519. 10.1186/s13063-017-2251-6 29110690 PMC5674846

[B18] Nunes R. d. M. Girão V. C. C. Cunha P. L. R. Feitosa J. P. A. Pinto A. C. M. D. Rocha F. A. C. (2021). Decreased Sulfate Content and Zeta Potential Distinguish Glycosaminoglycans of the Extracellular Matrix of Osteoarthritis Cartilage. Front. Med. 8, 612370. 10.3389/fmed.2021.612370 PMC8116584 33996844

[B19] Ogawa T. (2014). Ultraviolet Photofunctionalization of Titanium Implants. Int. J. Oral Maxillofac. Implants 29 (1), e95–e102. 10.11607/jomi.te47 24451893

[B20] Puelacher W. C. Mooney D. Langer R. Upton J. Vacanti J. P. Vacanti C. A. (1994). Design of Nasoseptal Cartilage Replacements Synthesized from Biodegradable Polymers and Chondrocytes. Biomaterials 15 (10), 774–778. 10.1016/0142-9612(94)90031-0 7986941

[B21] Ripmeester E. G. J. Timur U. T. Caron M. M. J. Welting T. J. M. (2018). Recent Insights into the Contribution of the Changing Hypertrophic Chondrocyte Phenotype in the Development and Progression of Osteoarthritis. Front. Bioeng. Biotechnol. 6, 18. 10.3389/fbioe.2018.00018 29616218 PMC5867295

[B22] Rizwan M. Hamdi M. Basirun W. J. (2017). Bioglass 45S5-Based Composites for Bone Tissue Engineering and Functional Applications. J. Biomed. Mater. Res. 105 (11), 3197–3223. 10.1002/jbm.a.36156 28686004

[B23] Rodrigues A. A. Batista N. A. Malmonge S. M. Casarin S. A. Agnelli J. A. M. Santos A. R. Jr. (2021). Osteogenic Differentiation of Rat Bone Mesenchymal Stem Cells Cultured on Poly (Hydroxybutyrate-co-hydroxyvalerate), Poly (ε-Caprolactone) Scaffolds. J. Mater. Sci. Mater. Med. 32 (11), 138. 10.1007/s10856-021-06615-6 34716801 PMC8557177

[B24] Shi Q. Qian Z. Liu D. Sun J. Xu J. Guo X. (2017). Maintaining the Phenotype Stability of Chondrocytes Derived from MSCs by C-type Natriuretic Peptide. Front. Physiol. 8, 143. 10.3389/fphys.2017.00143 28337152 PMC5340764

[B25] Tan Y. Chen D. Wang Y. Wang W. Xu L. Liu R. (2022). Limbal Bio-Engineered Tissue Employing 3D Nanofiber-Aerogel Scaffold to Facilitate LSCs Growth and Migration. Macromol Biosci. 1, e2100441. 10.1002/mabi.202100441 35020979

[B26] Thompson C. Plant J. C. Plant J. Wann A. Bishop C. Novak P. (2017). Chondrocyte Expansion Is Associated with Loss of Primary Cilia and Disrupted Hedgehog Signalling. eCM 34, 128–141. 10.22203/ecm.v034a09 28929469

[B27] Vinardell T. Sheehy E. J. Buckley C. T. Kelly D. J. (2012). A Comparison of the Functionality and *In Vivo* Phenotypic Stability of Cartilaginous Tissues Engineered from Different Stem Cell Sources. Tissue Eng. Part. A. 18 (11-12), 1161–1170. 10.1089/ten.TEA.2011.0544 22429262 PMC3360504

[B28] Wang C. Lin B. Qiu Y. (2022). Enhanced Hydrophilicity and Anticoagulation of Polysulfone Materials Modified via Dihydroxypropyl, Sulfonic Groups and Chitosan. Colloids Surf. B: Biointerfaces 210, 112243. 10.1016/j.colsurfb.2021.112243 34861540

[B29] Wright D. J. DeSanto D. J. McGarry M. H. Lee T. Q. Scolaro J. A. (2022). Nail Diameter Significantly Impacts Stability in Combined Plate-Nail Constructs Used for Fixation of Supracondylar Distal Femur Fractures. OTA Int. 5 (1), e174. 10.1097/OI9.0000000000000174 35187412 PMC8846389

[B30] Xue J. He A. Zhu Y. Liu Y. Li D. Yin Z. (2018). Repair of Articular Cartilage Defects with Acellular Cartilage Sheets in a Swine Model. Biomed. Mater. 13 (2), 025016. 10.1088/1748-605x/aa99a4 29125133

[B31] Xue K. Zhang X. Gao Z. Xia W. Qi L. Liu K. (2019). Cartilage Progenitor Cells Combined with PHBV in Cartilage Tissue Engineering. J. Transl Med. 17 (1), 104. 10.1186/s12967-019-1855-x 30925884 PMC6441183

[B32] Xue V. W. Ng S. S. M. Leung W. W. Ma B. B. Y. Cho W. C. S. Au T. C. C. (2018). The Effect of Centrifugal Force in Quantification of Colorectal Cancer-Related mRNA in Plasma Using Targeted Sequencing. Front. Genet. 9, 165. 10.3389/fgene.2018.00165 29868115 PMC5963087

[B33] Yang J. Gu C. Chen W. Yuan Y. Wang T. Sun J. (2020). Experimental Study of the Wettability Characteristic of Thermally Treated Shale. ACS Omega 5 (40), 25891–25898. 10.1021/acsomega.0c03258 33073114 PMC7557951

[B34] Yoon K.-H. Park J.-Y. Lee J.-Y. Lee E. Lee J. Kim S.-G. (2020). Costal Chondrocyte-Derived Pellet-type Autologous Chondrocyte Implantation for Treatment of Articular Cartilage Defect. Am. J. Sports Med. 48 (5), 1236–1245. 10.1177/0363546520905565 32125878

[B35] Yorseng K. Mavinkere Rangappa S. Parameswaranpillai J. Siengchin S. (2020). Influence of Accelerated Weathering on the Mechanical, Fracture Morphology, Thermal Stability, Contact Angle, and Water Absorption Properties of Natural Fiber Fabric-Based Epoxy Hybrid Composites. Polymers (Basel) 12 (10), 2254. 10.3390/polym12102254 PMC7600556 33008025

